# A novel generative adversarial networks modelling for the class imbalance problem in high dimensional omics data

**DOI:** 10.1186/s12911-024-02487-2

**Published:** 2024-03-28

**Authors:** Samuel Cusworth, Georgios V. Gkoutos, Animesh Acharjee

**Affiliations:** 1 Institute of Applied Health Research, University of Birmingham, Birmingham, UK; 2 NIHR Blood and Transplant Research Unit (BTRU) in Precision Transplant and Cellular Therapeutics, University of Birmingham, Birmingham, UK; 3 College of Medical and Dental Sciences, Institute of Cancer and Genomic Sciences, University of Birmingham, B15 2TT Birmingham, UK; 4 Institute of Translational Medicine, University Hospitals Birmingham NHS Foundation Trust, B15 2TT Birmingham, UK; 5 MRC Health Data Research UK (HDR), Midlands Site, UK; 6 Centre for Health Data Research, University of Birmingham, B15 2TT Birmingham, UK; 7 NIHR Experimental Cancer Medicine Centre, B15 2TT Birmingham, UK

**Keywords:** GAN, Multiomics, Class imbalance, Synthetic data

## Abstract

Class imbalance remains a large problem in high-throughput omics analyses, causing bias towards the over-represented class when training machine learning-based classifiers. Oversampling is a common method used to balance classes, allowing for better generalization of the training data. More naive approaches can introduce other biases into the data, being especially sensitive to inaccuracies in the training data, a problem considering the characteristically noisy data obtained in healthcare. This is especially a problem with high-dimensional data. A generative adversarial network-based method is proposed for creating synthetic samples from small, high-dimensional data, to improve upon other more naive generative approaches. The method was compared with ‘synthetic minority over-sampling technique’ (SMOTE) and ‘random oversampling’ (RO). Generative methods were validated by training classifiers on the balanced data.

**Supplementary Information:**

The online version contains supplementary material available at 10.1186/s12911-024-02487-2.

## Introduction

Many high-throughput omics studies involve differences in numbers of samples where one of the classes is higher in number compared to the other (e.g., number of healthy and number of disease samples) [1–3]. Usually, this number is more than three to fivefold and leads to class-imbalance, a common problem in omics studies. The prediction of future classes with class-imbalance poses a challenge. Some datasets, for example, ones related to cancer studies, include healthy samples that vastly outweigh the case samples, causing large class imbalances [4]. In technical terms, class imbalance is a difficult problem when training classifiers, causing the model to better learn the over-represented class. Many traditional and machine learning methods usually assume a balanced class setting for developing models, and a lack of such a setting results in specificity and sensitivity issues in data analysis. This has become more important in multi-omics data analysis and translational research [1], where, for example, associations between the biomarker and a disease result in a greater rate of false-negative and false-positive classifications [5]. A false-positive classification will result in an initial, greater expenditure of resources, assessing a positive outcome until the false-positive is confirmed, but a false-negative can have an overall greater negative impact [4].

In the literature, methods have been designed to deal with the class imbalance problem when training classifiers. Common approaches to class balancing work by reducing the sample size of the over-represented class (undersampling), increasing that of the under-represented class (oversampling or generation), or a mixture of both (hybrid) [4]. Sara Fotouhi et al. conducted an analysis of state of the art class balancing methods in cancer [4]. Results generally showed an increase in performance from oversampling techniques compared to undersampling techniques. Four different classification techniques were employed (repeated incremental pruning to produce error reduction, multi-layer perceptron, K-nearest neighbors, and C4.5, across 15 different cancer types, and the best performing balancing technique for each classifier-cancer pair, a mean area under the receiver operating curve (auroc) improvement of 8.6% compared to that without the use of balancing techniques. Generally, greater imbalances showed better performance with the balancing techniques.

Generative Adversarial Networks (GANs) [6] form an approach that learns the underlying distributions of the feature data space [7] catering to the ability to generate synthetic data. GAN is used in many aspects, including single cell genomics [8–10], RNA sequencing [11], and other omics studies for example metabolomics [12] already. The other application of GANs based techniques for addressing imbalance problems are already shown in the image data [13]. Some other examples are the meta analysis in the cancer image data [14]. Greater accuracy can be achieved over other popular methods, such as ‘synthetic minority over-sampling technique’ (SMOTE) and ‘random oversampling’ (RO) [4, 15], due to the learning of inter-feature relationships within the data.

In this study, we proposed a GAN-based methodology for use on high-dimensional data sets with small sample sizes, that allows for the synthesis of new samples that represent original data types. We further compared the performance of our approach against SMOTE and RO, auroc when using the data to train a classifier. We performed extensive simulations and applied the proposed methodologies on real world microarray and lipidomics data sets to demonstrate performance. We found evidence for an improved ability of the proposed GAN-based methodology to balance the classes of complex datasets with small sample sizes.

## Methods

### Generative adversarial networks

GANs are deep-learning-based, generative models that have grown in popularity in recent years [16]. GANs usually consist of two trained neural networks, a generator and a discriminator, with random noise classically used as input into the generator. The generator is trained to produce realistic (“fake”) data, from the noise input, to trick the discriminator, and the discriminator trained to distinguish between real and “fake” data. This resembles a zero-sum, non-cooperative game in game theory terms, hence the adversarial aspect to the architecture [17]. GANs have been widely applied to health research [7, 18, 19] generally demonstrating an improved performance, albeit at the cost of increased computational complexity, in generative tasks when compared to variational autoencoders and normalising flow models (other popular deep-learning, generative approaches) [20].

A GAN-based methodology is proposed here, that utilises a Wasserstein GAN with weight penalty (WGAN-WP) [21], alongside transfer learning and the addition of a distance metric to the generator loss function to increase variation in the generated results. We hypothesise that use of the proposed GAN, on high-dimensional data with small sample size, will allow for the generation of samples with greater representability than SMOTE and RO. This will allow for the generation of more accurate prediction models, using generation to balance classes of the data.

### GAN Architecture

**Fig. 1:**
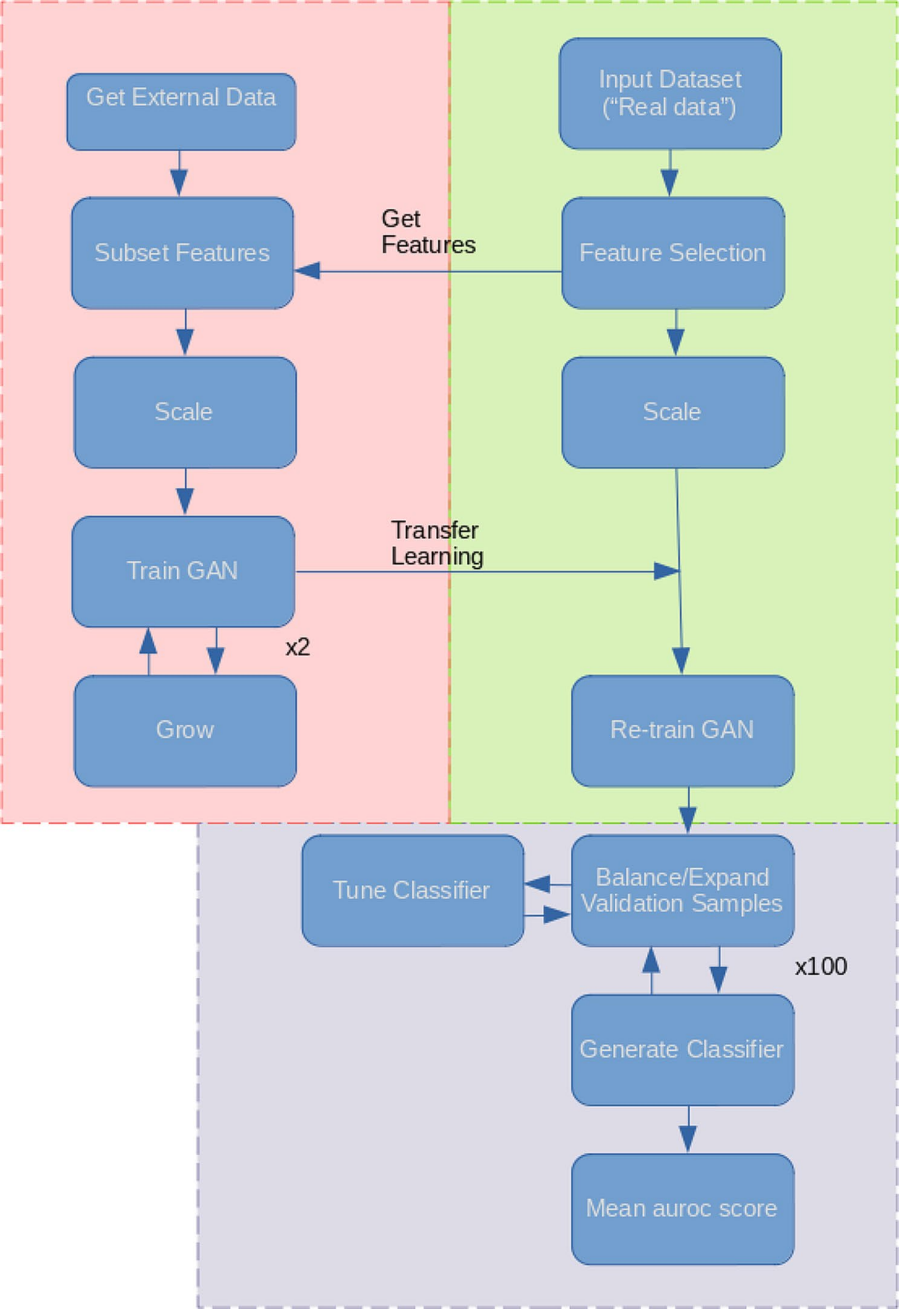
Study Workflow. Flowchart summarising the methodology of the study. Each of the 3 main parts of the study are highlighted in different colours. “Pre-training” is red, re-training is green, and validation is purple. Generative adversarial network (GAN); area under the receiver operating characteristic curve (auroc)

**Fig. 2:**
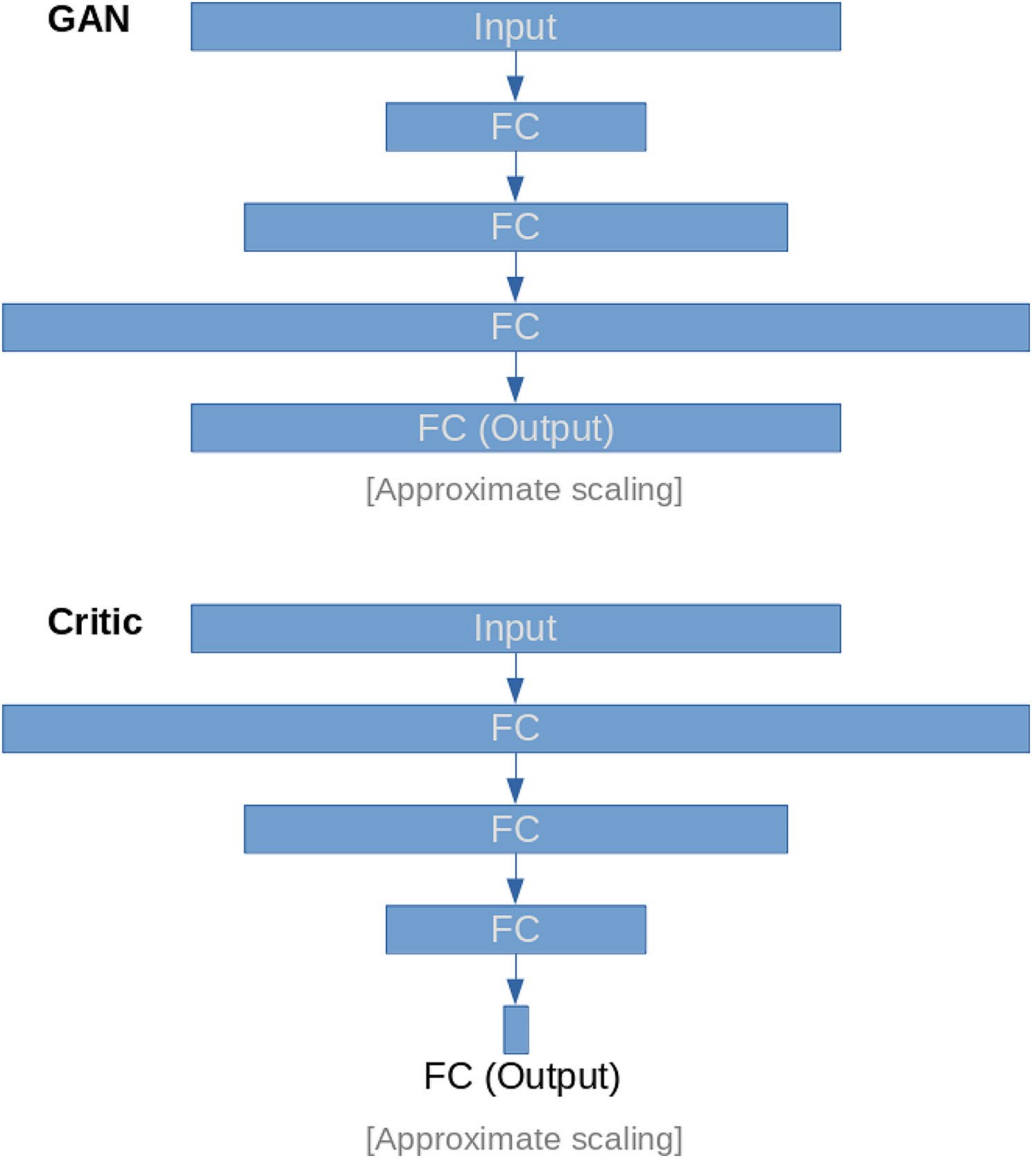
GAN Architecture Overview. Diagram showing the architecture of the neural network used as the GAN. Layers are displayed with approximate size scaling. Generative adversarial network (GAN); fully-connected layer (FC)

The workflow followed in this study is summarised in Fig. 1, and the general architecture of the proposed GAN is summarised in Fig. 2. The generator network layer sizes were defined as 50, 100, 200, with the input and output layers equal to the size of the input data. That of the critic was the same, but reversed, with the input layer equal to the size of the input data, and the output a single value (size 1).

#### Defining the loss function

A Wasserstein GAN was used over a classic GAN, where the Wasserstein distance is used to define the loss function, as opposed to binary predictions of ‘real’ or ‘fake’ [6]. Results have shown Wasserstein GANs to be highly effective at reducing key problems, such as mode collapse [6, 20]. Using two equal sized batches of input (noise and real), the critic loss was defined by the mean of the fake data prediction minus the mean of the real data prediction. A classic Wasserstein GAN would define the generator loss to be the mean of the fake data prediction from the critic multiplied by -1.

Due to the small sample size of the training data, the data would have a high likelihood of under-representing the true sample space. To prevent this, the log L2-matrix norm was calculated for the generated data during training, where a greater value would decrease the loss calculated. This was done to force the model to better cover the true sample space. A constant (alpha) was multiplied by the log L2-matrix norm to control the effect of this factor on the function, treated as a hyperparameter.

Where $$ {y}_{F}$$ and $$ {y}_{R}$$ are the fake and real predictions. $$ \left|{x}_{F}\right|$$ is the L2-matric norm of the generated data.

$$ Los{s}_{critic}=\overline{{y}_{F}}-\overline{{y}_{R}}$$

$$ Los{s}_{gen}=\left(-1\right)\left(\overline{{y}_{F}}\right)-\alpha \left|{x}_{F}\right|$$

Weight regularisation (α) was utilised during the training of the critic. It has been found that weight regularisation has a large effect on the mode of collapse of GANs [22]. Without regularisation, the critic tends to escalate the gradients during training, resulting in the critic learning a subset of samples well and the generator learning to generate samples that are not encompassed by the critic. Critic weight regularisation involves enforcing a Lipschitz constraint on the weights, limiting the gradients found to fall within set bounds. Our approaches adopted the WGAN-WP method, as described by Gulrajani et al. [21]. WGAN-WP generates a better gradient distribution across the Lipschitz bounds than classic Lipschitz constraints, providing more meaningful gradients to the loss function for training the generator.

#### Architectural considerations to improve training performance

To increase the ability of the generated GAN to extract effective features from limited data during training, transfer learning was utilised [23]. A dataset would be generated from an “external” dataset (no class labels) and used to train a GAN. The “external” data would consist of the same data modality as the “real” data, using the same features. Once sufficiently trained, the network would be retrained on the “real” data, to allow effective use of the network in the new context (a specific class). As such, the model was pre-trained to learn general features to extract from the modality of interest, giving the final network more power to learn effectively, than would be possible using raw input.

To allow for efficient training of the network, a progressive growing GAN was used [24]. The training involves the gradual addition of new layers, reducing the number of parameters to train at each epoch, which is important considering the small amount of data used for training.

The SELU activating function was used between each fully connected layer of the network to introduce non-linearity to the network, as recommended by [16]. This is a self-normalising activating function, preventing the need for batch normalisation between layers, reducing computational complexity. Use of this required specific weight-initialisation and dropout functions (kaiming normal and alpha-dropout respectively).

### Model validation

**Fig. 3:**
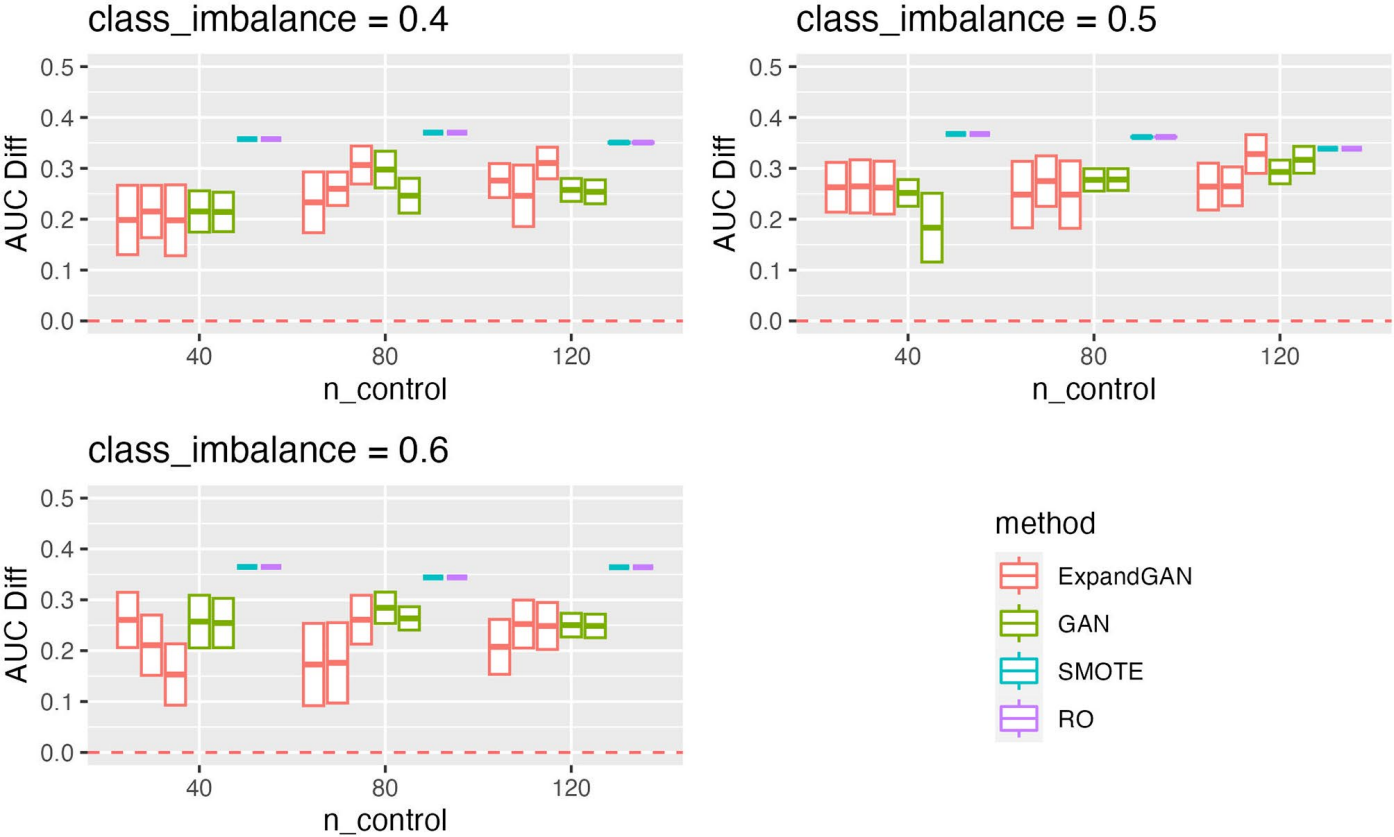
Simulation Validation Performance. Validation scores are summarised across experiments using the simulated data for GAN, ExpandGAN, SMOTE and RO methods. Boxes represent 1 standard deviation from the mean, and the horizontal line, of the box, the mean. The ‘AUC Diff’ defines the score difference between the 0.5 baseline (red, separated lines) and the validation score. Adjacent, same-coloured bars define results using different alpha hyperparameters, in order of 0/0, 1/0, 1/1 for ExpandGAN and 0 and 1 for GAN. Area under the receiver operating characteristic curve (AUC)

**Fig. 4:**
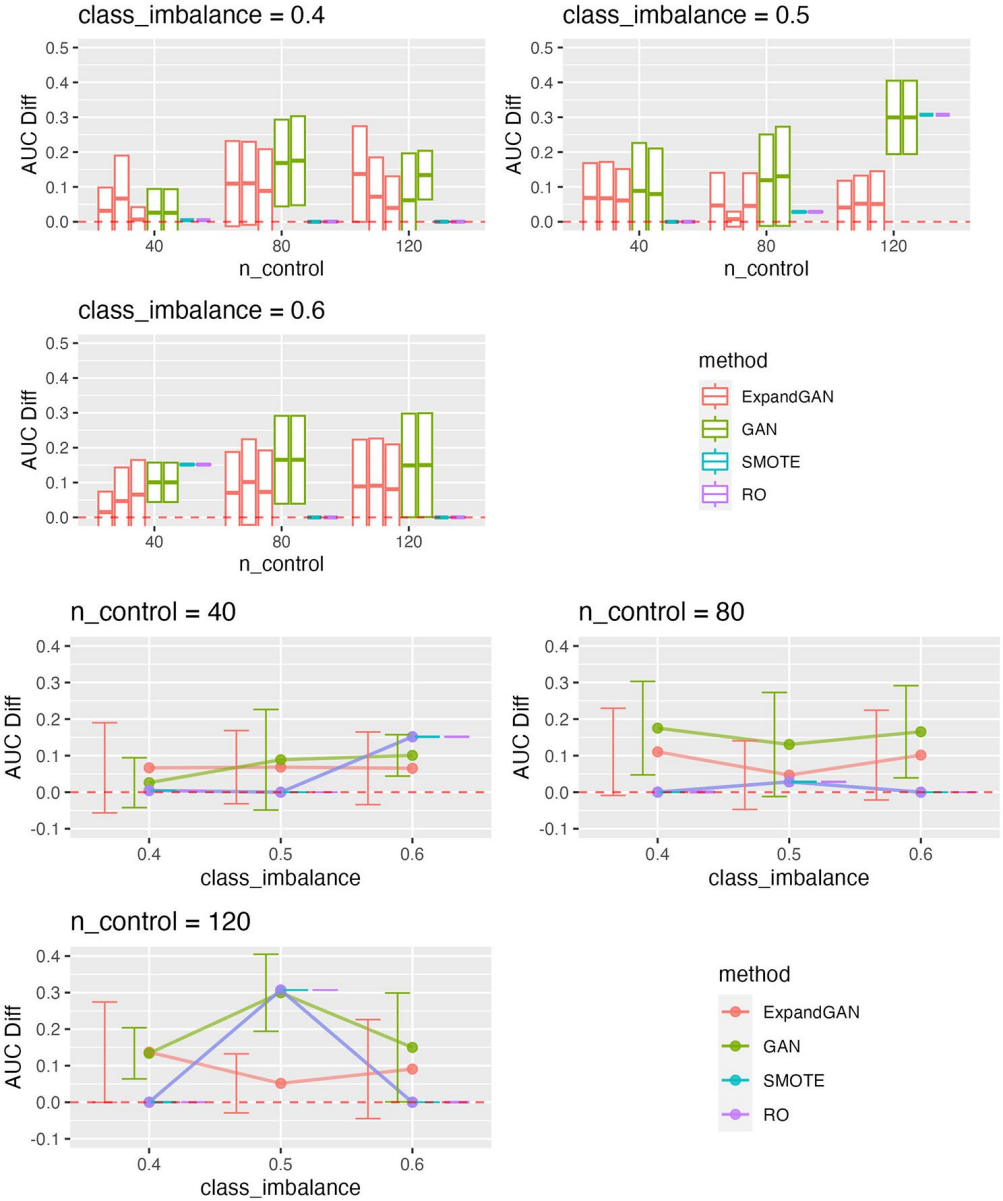
Microarray Validation Performance. Validation scores are summarised across experiments using the microarray data for GAN, ExpandGAN, SMOTE and RO methods. Boxes represent 1 standard deviation from the mean, and the horizontal line, the mean. The ‘AUC Diff’ defines the score difference between the 0.5 baseline (red, separated lines) and the validation score. Adjacent, same-coloured bars define results using different alpha hyperparameters, in order of 0/0, 1/0, 1/1 for ExpandGAN and 0 and 1 for GAN. Where/if SMOTE varied across experiments with changed alpha, the results are shown in multiple adjacent bars. Area under the receiver operating characteristic curve (AUC)

**Fig. 5:**
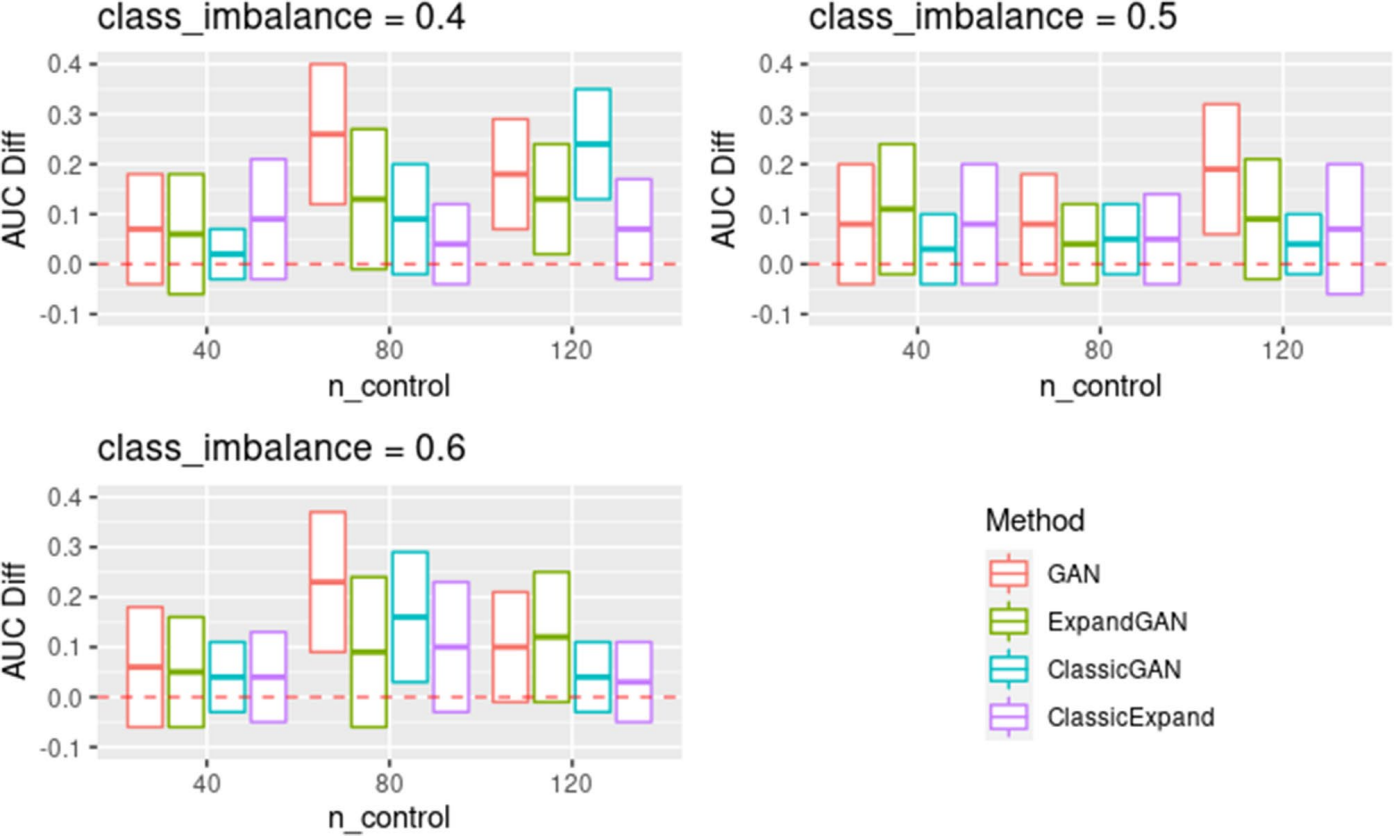
Classic WGAN-WP Comparison Results. Validation scores are summarised across experiments using the microarray data for GAN, ExpandGAN, ClassicGAN and ClassicExpand methods. Boxes represent 1 standard deviation from the mean, and the horizontal line, of the box, the mean. The ‘AUC Diff’ defines the score difference between the 0.5 baseline (red, separated lines) and the validation score. GAN and ExpandGAN results shown are those using the alpha hyperparameter combination resulting in the greatest mean validation score. Area under the receiver operating characteristic curve (AUC); Wasserstein generative adversarial network with weight penalty (WGAN-WP)

**Fig. 6:**
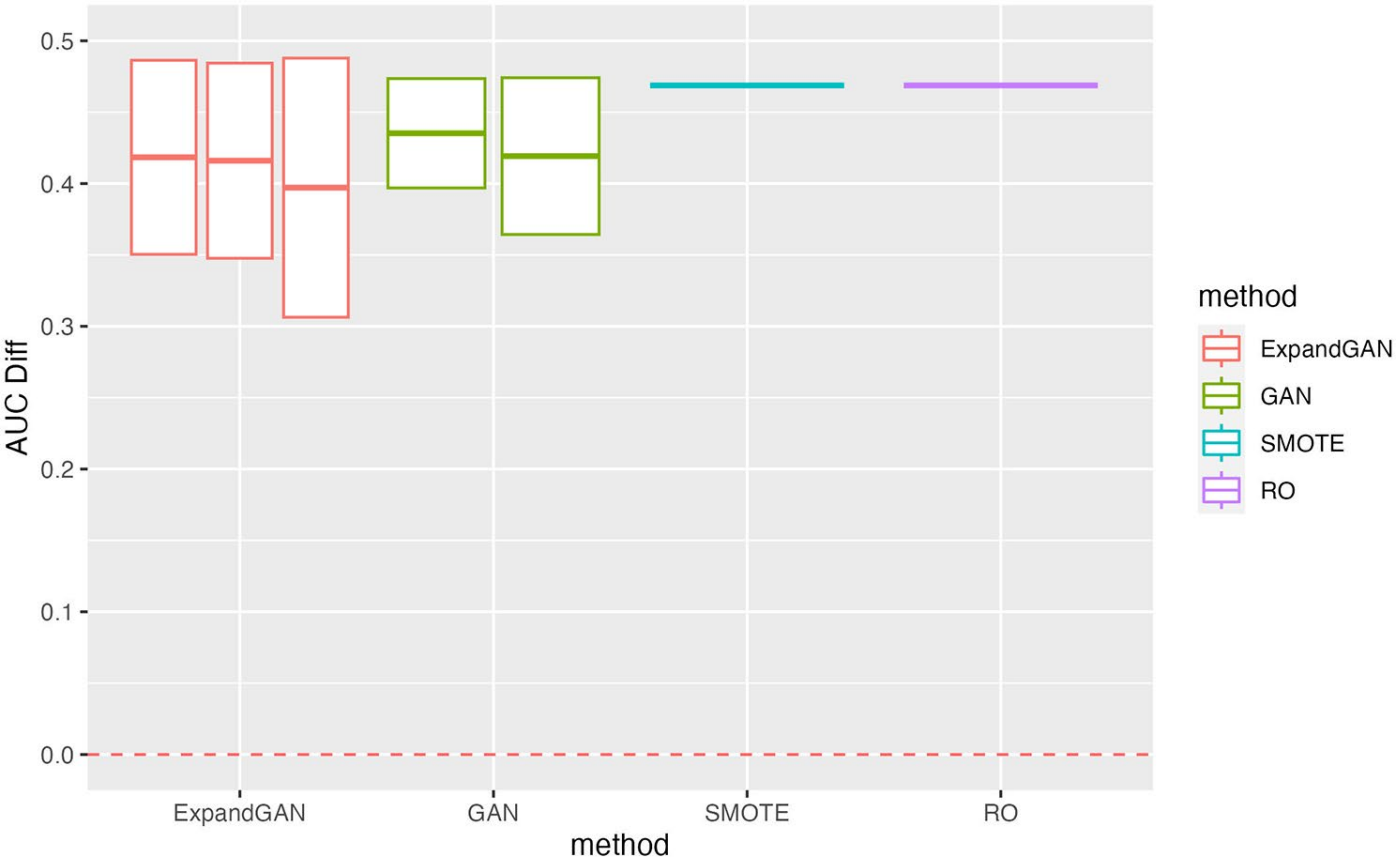
Lipidomics Validation Performance. Validation scores are summarised across experiments using the lipidomics data for GAN, ExpandGAN, SMOTE and RO methods. Boxes represent 1 standard deviation from the mean, and the horizontal line, of the box, the mean. The ‘AUC Diff’ defines the score difference between the 0.5 baseline (red, separated lines) and the validation score. Adjacent, same-coloured bars define results using different alpha hyperparameters, in order of 0/0, 1/0, 1/1 for ExpandGAN and 0 and 1 for GAN. Area under the receiver operating characteristic curve (AUC)

To assess the ability of the trained GAN to improve the performance of prediction models, by balancing classes through generation, the validation method proposed by Huang et al. was employed [16]. Training data and test data were assumed to the “real” data and “validation” data respectively. Data was balanced through generation using GAN, SMOTE and RO. GAN was also used to increase the sample sizes of both classes, termed ‘expanded generation’, which was employed so as to double the number of samples of the over-represented class, and balance this in the under-represented class. The balanced data was then used to train a classifier, using the ‘HistGradientBoostingClassifier’ method from Scikit-learn [25]. Five-fold cross validation was used to tune the hyperparameters. For the HistGradientBoostingClassifier, learning rates of 10^− 4^, 10^− 3^, 10^− 2^, 1 and 10, and minimum samples per leaf values of 1, 10, 25, 50, 75 were used in tuning (other parameters were left as default). 100 classifiers were produced, using the corresponding hyperparameters, generating new data (balancing) for each iteration. Mean auroc scores (with standard deviation) were computed. We defined the score difference between the 0.5 baseline and the validation scores (these values are reported in Figs. 3, 4, 5 and 6).

Where GAN and ExpandGAN were employed, the alpha combination that resulted in the greatest mean auroc was used to compare to other methods. The effects of different values of alpha were analysed separately.

Differences in auroc, across each set of experiments (simulated, microarray, or lipidomics), were tested for significance using Welch’s t-test (BSDA R package [26]), adjusting p-values using the Benjamini-Hochberg method.

Additionally, a classic WGAN-WP [21] was built using the same methodology as the proposed GAN method to assess the effect of the pre-training procedure. The classic WGAN-WP was trained with no distance penalty to the generator loss (alpha = 0), no pre-training loop, and no growing (all layers were initialised together).

### Data acquisition

#### Data simulation

For the simulated data, the ‘Umpire’ R package [27] was used to simulate a microarray dataset. Two clusters were simulated with 150 features, representative of the feature space after feature selection of the microarray data (described below). Noise from the gamma distribution (default) was added to the features (shape = 2, scale = 0.5). 700 samples were used for validation and 300 as the “external” dataset. The “external” dataset consisted of a mixture of the two clusters, without labels. The number of samples of each cluster was changed for each experiment, altering the number of control samples and the level of class imbalance (Table 1). The same “external” and validation data were used across all experiments, but each experiment used different generated “real” data.

**Table 1:** Experiment input data summary

Experiment ID	Number of control samples	Class imbalance
1	40	0.4
2	80	0.4
3	120	0.4
4	40	0.5
5	80	0.5
6	120	0.5
7	40	0.6
8	80	0.6
9	120	0.6

Table summarising the number of control samples and class imbalance for each experiment. Class imbalance is multiplied by the number of control samples to define the number of samples in the other class

#### Public omics data sets

We have obtained the following public data sets from the published papers and the Gene Expression Omnibus (GEO) database [28]. A detailed information of the data listed in the Table 2.

**Table 2:** Hyperparameter Summary

id	func_optim	beta	batch_size	iter_critic	dropout_prob	gen_structure	critic_structure	max_epochs_1	max_loss_1	n_epochs_2	lr_1	lr_2	rate_save	diff_epochs	max_loss_2	min_loss_2	instab_constant
lipid_pretraining	SGD	13	30	4	0.5	50, 100, 200	200, 100, 50	500	3	1000	0.0005, 0.0001, 0.001	1e-05, 5e-05, 0.0001	20	500	15	-10	3
lipid_retraining	SGD	13	10	2	0.7	50, 100, 200	200, 100, 50	500	3	5000	0.0005, 0.0001	1e-05, 5e-05	10	500	15	-15	3
micro_pretraining	SGD	13	30	4	0.5	50, 100, 200	200, 100, 50	500	3	1000	0.0005, 0.0001, 0.001	1e-05, 5e-05, 0.0001	20	500	15	-10	3
micro_retraining	SGD	13	10	2	0.7	50, 100, 200	200, 100, 50	500	3	5000	0.0005, 0.0001	1e-05, 5e-05	10	500	15	-15	3
sim_pretraining	SGD	13	30	4	0.5	50, 100, 200	200, 100, 50	500	3	1000	0.0005, 0.0001, 0.001	1e-05, 5e-05, 0.0001	20	500	15	-10	3
sim_retraining	SGD	13	10	2	0.7	50, 100, 200	200, 100, 50	500	3	5000	0.0005, 0.0001	1e-05, 5e-05	10	500	15	-15	3

Table showing hyperparameter values used across the experiments for each dataset. Pre-training and re-training values are shown for each experiment. Iter_critic defines the number of critic training iterations before 1 training cycle of the generator. n_epochs defines the number of epochs trained, using the determined learning rates. Instab_constant was used to define the effect of instability when determining the most effective learning rates. Furthermore, max_loss_1 and max_epochs_1 were used to define this in the first training iterations, and max_loss_2, min_loss_2 in the second. diff_epochs defines the max number of epochs to train when determining this.Stochastic gradient descent (SGD), metabolomics study (lipid), microarray study (micro), simulated microarray study (sim), optimising function (func_optim), instability (instab), probability (prob), iter (iterations), learning rate (lr)

#### Microarray data sets

In this study we have used public Hepatocellular carcinoma microarray data set. Microarray technology [29] is an efficient tool employed in molecular biology and genomics to examine the expression of many genes simultaneously. Gene expression [30] levels in a specific sample, which offers valuable information about the functioning of genes in different conditions (for example: cancer vs. non cancer) or tissues (for example: mucosal tissue vs. tumour tissues). Microarray data set were obtained from the GEO database [28]. GSE14520, GSE25097, and GSE36376 accessions were used for “external” “validation,” and “real” data, respectively [31]. Datasets were filtered for all cancer samples and adjacent, non-cancer samples. The ‘GEOquery’ R package was applied to obtain the datasets [32], using the sample accessions obtained from the Entrez Programming Utilities [33]. The “real” data was randomly sampled to obtain the corresponding number of control samples and class imbalance for each experiment (Table 1). The gene ontology and pathway analyses were conducted on selected features (explained below) from the “real” data, using ‘EnrichR’ [34–36].

#### Lipidomics data sets

Lipids data can be generated through various experimental and analytical techniques, as lipids are a diverse group of molecules that include fats, oils, phospholipids, and steroids. We have performed direct infusion high-resolution mass spectrometry (HRMS) method with plasma/serum samples to generate lipids. Multiple lipidomics data sets were obtained from previously published Cambridge Baby Growth Study (CBGS) cohorts [37]. Lipidomic profiles were compared between infant groups who were either exclusively breastfed, i.e., human milk (HM), exclusively formula-fed or mixed-fed at various levels. The three datasets considered were CBGS_1, CBGS_2 and Pregnancy Outcome Prediction Study (POPS), used for the “real”, “external,” and validation datasets, respectively. All available lipidomics features were used. “real” and “validation” sets were filtered for ‘Formula’ and ‘HM’ classes, for binary classification. “External” data used both the above classes and ‘HM & Formula” (all classes available).

### Model training

Our training approach is summarised in Fig. 1. First, regarding the simulated and public microarray datasets, the data underwent feature selection, reducing the feature space to ∼ 150 features. Differential gene expression analysis for the microarray data was performed prior to this study [31]. The top 200 genes, by decreasing Benjamini-Hochberg adjusted p-value (adj.P), were selected from the “real” differential gene expression results. The genes were then filtered to remove any genes, across the three datasets, that had any missing values across all three, leaving 135 genes. The simulation data was simply simulated with 150 samples, representing a dataset after feature selection.

Two ‘ColumnTransformers’ were then built using the ‘Scikit-learn’ Python package [25], one for the “external” data and one for the “real” data. The column transformers consisted of a robust scaler and a power transformer (Yeo-Johnson method) [25]. Columns would automatically be designated one of the two scaling methods, using the ‘skewtest’ method (‘Scipy’ Python package) [38]. A significant difference would be assessed between the distribution of each feature and the normal distribution, and if significant (p < 0.05), the power transformer would be used, otherwise, the standard scaler would be used. “External” and “real” datasets were then scaled using the corresponding ‘ColumnTransformers’. The"external” transformer was built using 200 random samples from the “external” data.

A GAN was then trained on the “external” data. Fully-connected layers were added progressively to the network, training the network before adding a new layer. Once trained, transfer learning was conducted, re-training the network on the “real” data. Two GANs would be produced here, both using the pre-trained weights and biases as a base before re-training. Each GAN would be re-trained on one class of the “real” data.

GANs necessitate, due to convergence difficulties, being assessed frequently throughout training to ensure the best performing parameters are used in the training procedure [16]. The GAN results were assessed at regular epochs (20 epochs), using the loss-values to assess the model. An early-stopping-like method was used, specifying a large number of epochs to train, and selecting an optimal epoch. Each epoch was selected by first removing all values past the 1st instance of a critic loss > 15 or <-10. An objective function was then fitted to the curve using the ‘curve_fit’ method of scipy [38]. The ‘KneeLocator’ method (kneed package) [39] was then used to find the elbow point of the curve generated from the objective function.

To reduce the effect of mode collapse on the network, higher dropout probabilities, with smaller learning rates, were used on the GAN layers during transfer learning, due to the small amount of data used.

The hyperparameters for the re-training loops were tuned using an automated method. A larger initial learning rate (lr_1) was used, followed by a smaller learning rate (lr_2). Learning rates of 0.0005, 0.0001 and 0.001 were assessed for lr_1 and 0.00001, 0.00005 and 0.0001 for lr_2. Lr_1 was trained to a maximum of 5000 epochs, and the learning rate resulting in the smallest critic loss above 3 was used. Lr_2 was trained for 5000 epochs, and a function was defined to balance speed and stability of training. First, the first instance of a critic loss of > 15 or <-10 was removed. The apexes were then found across this curve (representing the noise), and the difference between the corresponding apexes was calculated. Instability was defined by the mean of the top 50 greatest differences. The speed of training was defined by the difference between the mean of the 1st 500 epochs and the last 500 epochs of the curve. The lr_2 that resulted in the smallest ‘3*instability - difference’ was selected.

The batch sizes used were 10 and 20 for the underrepresented, overrepresented classes, respectively. The pre-training used a batch size of 30.

The effect of the alpha hyperparameter, controlling the effect of the log L2-matrix norm in the generator loss function, was assessed with different values. Regarding the under-represented class GAN and the over-represented class GAN respectively, values of 1/1, 1/0 and 0/0 were used. Therefore, for each experiment, three sets of results would be produced (one for each set of alpha values). Alpha was set at 0 during the pre-training loops.

### Variable number of samples for expandGAN

The effect of number of generated samples, past the number of samples of the overrepresented class, on the performance of a predictor model was assessed. The number of samples to generate was determined by multiplying the number of samples of the overrepresented class by a “multiplier” value, adding this to the number of samples of the overrepresented class, and generating samples until both classes had reached this number. The “multiplier” values used were 0.25, 0.5, 0.75 and 1.0, where 1.0 would double the number of samples of the overrepresented class.

### Feature selection using regularization methods

Feature reduction was carried out using two feature selection techniques, namely Least Absolute Shrinkage and Selection Operator (LASSO) [40], and Elastic Net [41]. These regularization methods automatically identify significant variables by reducing the coefficients of irrelevant predictors to zero, achieving a sparse representation. To implement biologically relevant feature selection using the LASSO and Elastic Net (EN) algorithms, we systematically optimized the penalty parameter associated with each method in an unbiased manner. This involved randomly dividing the samples into a training set, comprising 75% of the total samples, and a test set with the remaining 25% of samples. A 10-fold cross-validation was then conducted on the training set (inner loop set) to determine an optimized penalty parameter that could be employed in the LASSO and EN models. Mathematically, both LASSO and EN models can be characterized using a single penalty function denoted as “α. We performed a stability analysis i.e iterated the process multiple times and derived important features based on frequency over 10 iterations. We took the top 25% of the features that appeared in both EN and LASSO and used them for further downstream analysis [42]. We have used regularization methods using DEG genes and the all the lipids features.

For the experiments using lasso feature selection, the same pipeline was used, as was used in the corresponding analyses above (including statistical testing).

### Scripts and tools

R [43] and Python [44] were used to build the scripts, using R-studio and Spyder integrated development environments, respectively [45, 46]. The GAN methods used were built and trained using the ‘Pytorch’ Python package [47]. Models were trained using either a 6th generation Intel® Core™ i5 CPU, RTX 2060 super GPU, and 24 GB RAM, or an M2 Macbook pro, 8 GB RAM. Both used a solid state drive to store model outputs. Where time taken to train noticeably changes across experiments, this may be due to the different machines used to train the models.

## Results

### Training results

Overall, the pre-training took 1,815 s for 15,500 epochs, and 1,742 s for 15,000 epochs for simulation and microarray data, respectively. Regarding re-training, across the microarray experiments (not including classic WGAN-WP), GANs were trained at a mean of 471 s, per GAN. Across the simulation experiments, a mean of 280 s.

#### Hyperparameter tuning

The hyperparameters used to train the models are summarised in Table 3. Due to pre-training showing greater instability upon adding the third layer, the critic: generator ratio was changed to 2:1, and dropout probability to 0.7, to help stabilise training. This dropout probability and ratio was maintained throughout re-training.

**Table 3:** Datasets Summary

Data set	Number of the case and control samples	Number of the features (after selection)	Reference	
GSE14520	225, 220	135	Roessler S, Jia HL, Budhu A, Forgues M et al. A unique metastasis gene signature enables prediction of tumor relapse in early-stage hepatocellular carcinoma patients. Cancer Res 2010 Dec 15;70 [24]:10202-12. PMID: 21,159,642	
GSE25097	268, 243	135	Tung EK, Mak CK, Fatima S, Lo RC et al. Clinicopathological and prognostic significance of serum and tissue Dickkopf-1 levels in human hepatocellular carcinoma. Liver Int 2011 Nov;31 [10]:1494 − 504. PMID: 21,955,977	
GSE36376	240, 193	135	Lim HY, Sohn I, Deng S, Lee J et al. Prediction of disease-free survival in hepatocellular carcinoma by gene expression profiling. *Ann Surg Oncol* 2013 Nov;20 [12]:3747-53. PMID: 23,800,896	
Cambridge Baby Growth Study (CBGS) cohorts	CBGS-1 85 HM, 87 FM; CBGS-2 43 HM, 25 FM, 27 HM + FM; POPS 16 HM, 11 FM	218	Acharjee, A., Prentice, P., Acerini, C. et al. The translation of lipid profiles to nutritional biomarkers in the study of infant metabolism. Metabolomics 13, 25 (2017). 10.1007/s11306-017-1166-2	

Summary of public datasets used in this study. Reference refers to the earliest citation present on the corresponding NCBI Gene Expression Omnibus [28] page. Number of features reflects the input data to the corresponding GAN model

### Classification performance

#### Results comparison

The classification performances differed largely between the simulation and microarray datasets. The best results among different combinations of alpha value for each experiment across each method, are summarised in supplementary Fig. 1. Generally, the simulation results show that the classification methods managed to identify some patterns that enabled distinguishing between different classes (Fig. 3). All mean auroc scores were > = 0.70. The microarray experiments showed reduced classification performances compared to the simulation results, where all but 1 SMOTE/RO experiments scored < = 0.65. All ExpandGAN results were similarly < 0.65, but GAN results showed 4 experiments where auroc > = 0.65. This data showed greater distinctions between the SMOTE/RO and GAN-based methods. The microarray experiments showed greater variances than the simulation experiments, ranging from 0 to 0.15, and 0 to 0.05 respectively (Figs. 3 and 4). All microarray proposed GAN-based methods reported standard deviations > = 0.06, greater than the range of SMOTE/RO (all 0).

#### Simulation results

The results obtained across the simulation experiments (Fig. 3), showed that for 40 and 80 control samples, SMOTE/RO outperformed the GAN-based methods, with all differences being significant. With 120 samples, GAN (not ExpandGAN) showed closer performance to SMOTE/RO, with an average difference of 0.076 (SMOTE/RO performing better). Despite this, SMOTE/RO¬ auroc remained significantly greater.

#### Public microarray datasets

The results obtained across the microarray experiments are shown in Fig. 4. All reported GAN auroc scores were significantly different from both SMOTE and RO, apart from the experiment using 120 control samples with class imbalance 0.5. GAN performed significantly better than SMOTE/RO in 7 of these 8 experiments. These improved differences ranged from 0.02 to 0.18, with a mean of 0.12. GAN and SMOTE/RO reported 0.05 difference in mean auroc in the insignificant experiment, although GAN did have greater standard deviation of 0.11, compared to 0. SMOTE/RO significantly outperformed GAN with 40 samples and 0.6 class imbalance, 0.05 difference. Generally, ExpandGAN performed worse than GAN. All differences were significant, apart from 120 controls and 0.4 class imbalance, and 40 controls and 0.5 class imbalance. Out of the significant results, GAN outperformed ExpandGAN in all but the experiment with 40 control samples and 0.4 class imbalance. When analysing the validation results for the case of classic WGAN-WP, ‘ClassicGAN’ methods were compared with GAN, and ‘ClassicExpand’ methods with ‘ExpandGAN’ (Fig. 5). ‘ClassicGAN’ results ranged from 0.74 to 0.52, and ‘ClassicExpand’ 0.60 to 0.53. This compares to 0.76 to 0.56 and 0.63 to 0.54 for GAN and ExpandGAN respectively.

Differences between GAN and ClassicGAN were significant in all experiments apart from experiment 7. In all but one of these experiments (experiment 3), mean auroc scores of GAN exceeded that of ClassicGAN. These differences ranged from 0.17 (experiment 2) to 0.03 (experiment 5), mean 0.08, and ClassicGAN exceeded GAN by 0.06 in experiment 3. Differences between ExpandGAN and ClassicExpand were less significant, but significance was found in experiments 2, 3 and 9, where mean auroc scores of ExpandGAN exceeded that of ClassicExpand. These differences ranged from 0.09 (experiments 2 and 9) to 0.06 (experiment 3). This part of the analysis (comparison with ClassicGAN ClassicExpand) was run on an older version of the pipeline. This version only implemented the ‘HistGradientBoostingClassifier’, but due to the better performance of this method over SVC, when using the GAN-based methods, we did not predict this to make any significant difference to the results.

#### Effect of the distance metric on the generator loss

The effects of altering the contribution of the distance metric to the generator loss varied across the experiments (Figs. 3 and 4). Overall, two different values of alpha (the constant multiplied by the distance metric) were assessed for both the under-represented and over-represented classes (1 and 0), but the combination of 0/1 for under-represented, over-represented respectively, was not assessed.

Regarding the simulation data with the ExpandGAN method, the most notable differences, when using different combinations of this hyperparameter occurred when using 0.6 class imbalance with 40 and 80 controls samples, 0.4 class imbalance with 80 and 120 control samples, and 0.5 class imbalance with 120 control samples. In all but one of these cases, an increase in performance can be seen when utilising this, and the other a decrease in performance. This hyperparameter had minimal effect on the experiments using the GAN method, although some evidence of decreased performance can be seen with 80 controls, 0.4 class imbalance, and 40 controls, 0.5 class imbalance.

Regarding the microarray data, altering alpha for the under-represented class, generally resulted in little difference when using the GAN method. One more noticeable result happened when using 120 controls with 0.4 class imbalance, where altering alpha increase the performance, with reduced standard deviation. When using the ExpandGAN method, the hyperparameter had little effect on performance, where most differences consisted of a minor change in mean and standard deviation. A couple of noticeable differences can be seen with 40 control samples and 0.4 class imbalance, and 80 samples and 0.6 class imbalance, where altering alpha had a noticeable effect on the standard deviation. 120 controls with 0.4 class imbalance was also noticeable, although showing reduced performance when altering alpha.

#### Public lipidomics datasets

The lipidomics results (Fig. 6) were compared in the same way as with the other datasets, where the greatest mean auroc score for each experiment and each method (resulting from different combinations of alpha) were used for comparison. All methods were significantly different from each other, apart from SMOTE vs. RO, and all methods reported mean auroc of > 0.89. ExpandGAN mean auroc was significantly smaller than all other methods and GAN smaller than SMOTE/RO (by 0.03). The inclusion of alpha (the distance metric) made little difference to the results, although improving the standard deviation of the GAN method (reduced).

### Gene ontology and pathway enrichment

The microarray data Gene Pathway (GP) enrichment results are provided in the supplementary Tables 1 and 2. The results show genes associated with hepatocellular carcinoma, such as replication and transcription [48], and zinc homeostasis [49].

### Variable number of samples for expandGAN

The effect of increasing the number of additional samples generated by the ExpandGAN method was analysed. Across the public, microarray experiments, the results showed wide standard deviation of the auroc scores, therefore there is unlikely to be any significance between altering the number of additional samples generated. Where there were smaller standard deviations found, the auroc scores found were low. Across the simulation experiments, the standard deviations found were smaller than the public, microarray experiments. There was limited evidence to suggest a general trend in the performance of the method when altering the number of additional samples generated. Despite this, the HistGradientBoostingClassifier experiments with 120 samples and class imbalance of 0.6 showed some evidence of increasing performance, with increased numbers of samples generated. In the experiment not using alpha (alpha = 0), auroc of multiplier of 0.25 was 0.72 (0.05 standard deviation), and multiplier of 1.0 was 0.84 (0.03 standard deviation).

### Effect of the feature selection on the data sets

**Fig. 7:**
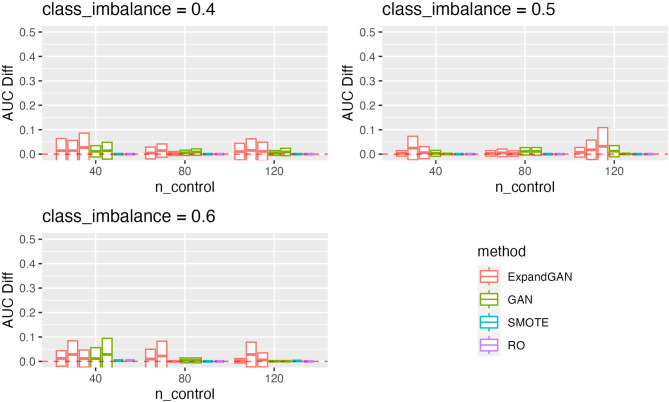
Features Selected Microarray Data Validation Performance. Validation scores are summarised across experiments using the microarray (LASSO and EN feature selection) data for GAN, ExpandGAN, SMOTE and RO methods. Boxes represent 1 standard deviation from the mean, and the horizontal line, the mean. The ‘AUC Diff’ defines the score difference between the 0.5 baseline (red, separated lines) and the validation score. Adjacent, same-coloured bars define results using different alpha hyperparameters, in order of 0/0, 1/0, 1/1 for ExpandGAN and 0 and 1 for GAN. Where/if SMOTE varied across experiments with changed alpha, the results are shown in multiple adjacent bars Area under the receiver operating characteristic curve (AUC).

**Fig. 8:**
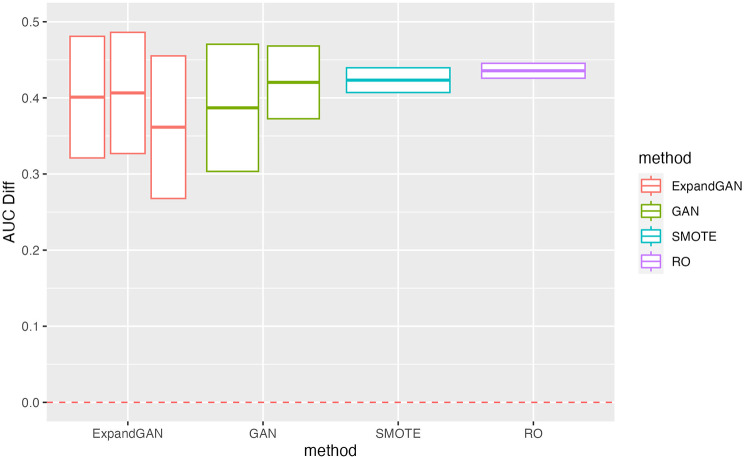
Features Selected Lipidomics Data Validation Performance. Validation scores are summarised across experiments using the lipidomics (LASSO and EN feature selection) data for GAN, ExpandGAN, SMOTE and RO methods. Boxes represent 1 standard deviation from the mean, and the horizontal line, of the box, the mean. The ‘AUC Diff’ defines the score difference between the 0.5 baseline (red, separated lines) and the validation score. Adjacent, same-coloured bars define results using different alpha hyperparameters, in order of 0/0, 1/0, 1/1 for ExpandGAN and 0 and 1 for GAN. Area under the receiver operating characteristic curve (AUC).

We used selected features from both microarray and lipidomics to investigate the performance of the model. When comparing the results described above to the same analysis but using LASSO and EN feature selection on the “real” data to select input features, final classification performances were varied. When using the lipidomics data (Fig. 7), RO significantly outperformed all other methods. The rest of the methods did not significantly deviate from each other. GAN methods showed greater standard deviation around auroc scores. All mean aurocs (of the results used in the statistical tests) were above 0.9. The microarray results (Fig. 8), showed poor performance across all methods, with all mean auroc scoring less than 0.6.

The number of selected features was 33 and 34 for microarray and lipidomics respectively; much less compared to the number of features used in the main analysis. roc curves for these experiments are shown in supplementary Figs. 6 and 7 (microarray, lipidomics respectively).

## Discussion

In this research, we introduced a methodology based on GANs designed for high-dimensional datasets with limited sample sizes. In the realm of high-dimensional data analysis, GANs play a pivotal role by tackling issues associated with limited data availability, intricate distributions, and the necessity for efficient representation learning. This methodology enables the generation of new samples that accurately capture the characteristics of the original data types. A brief overview of the structure of the GAN method can be found in Fig. 2 with the generator having layers of sizes 50, 100, 200, and input/output layers matching the input data size. The critic, part of the GAN, had reversed layer sizes. Instead of a classic GAN, we used a Wasserstein GAN, employing the Wasserstein distance for the loss function, which helps address issues like mode collapse.

To date, imbalanced classes remain a great challenge for the application of machine/deep-learning methods, leading to an over-representation in the trained models [4]. This is problematic when using high-dimensional data, especially when only a small number of samples are available, where many balancing techniques, as well as the few data perturbing methods that can be used with high-dimensional data, fail to allow effective generalisation of the classes [50]. When using the SMOTE/RO methods in this study, with the limited microarray data, the majority of experiments resulted in poor performance of the final trained classifiers, supporting this. Generative methods, using deep-learning techniques, have shown promising results in generalising over the underlying training data, potentially allowing for improvements in increasing the generalisability of the cancer samples in cancer analysis [18, 19, 51]. Unfortunately, deep-learning, generative methods require large numbers of samples for effective training, with more samples required with higher dimensional data, which is quite challenging when considering certain datasets, such as microbiota related ones [52]. GANs have typically been trained on data with regional dependencies (e.g., image, brain connectome, interaction networks), or lower dimensional data [18, 19, 51] whereas their application across higher dimensional data with small sample sizes, for fully generative purposes, is limited. We therefore proposed a preliminary design of a GAN architecture that aimed to improve on these limitations.

The main drawbacks of GANs are their increased complexity, compared to other methods mentioned [20]. The use of two separate networks in training GANs leads to increased computational complexity, with characteristically difficult convergence [22]. GANs also suffer from mode collapse, where the generator learns an effective output to “trick” the discriminator, limiting the generalisability of the data generated. Modern variations of GANs have reduced these key problems [20], but care is still taken to avoid them (as described in the methods section).

### Simulation study

The simulation datasets reported significantly greater performance of SMOTE/RO over both GAN-based methods, across all experiments. Considering the results seen across the microarray experiments, it has been hypothesised that the simulation data generated did not fully represent the complexity of real, microarray data.

### Public data sets

#### Microarray data sets

The microarray data contrasted the results of the simulation data, showing in all but 2 experiments a significant increase in performance of GAN over SMOTE/RO. To note, the GAN reported mean auroc scores deviated much more than SMOTE/RO. ExpandGAN did not report any significant benefit over use of GAN, with evidence to suggest poorer performance when using ExpandGAN.

When comparing classic GAN-based methods to the proposed GAN-based methods, GAN was reported to significantly outperform ClassicGAN in the majority of experiments (7 out of 9), with 1 experiment showing no significant difference, and 1 ClassicGAN outperforming GAN. In the experiments where GAN significantly outperformed ClassicGAN, a mean difference between scores of 0.08 was reported, therefore showing a meaningful increase in performance. The differences between ExpandGAN and ClassicGAN were not found to be as meaningful, but in the minority of experiments where a significant difference was found (3 out of 9), ExpandGAN was found to outperform ClassicExpand, with differences in scores ranging from 0.09 to 0.06, showing some evidence of an improvement with the proposed method over a classic WGAN-GP here.

To note, these methods were performed with an earlier implementation of the scripts. These scripts were very similar, but differed mainly in the use of the SVC classifier, in addition to the histGradientBoosting classifier. Due to the better performance seen using the histGradientBoosting classifier over the svc in the other experiments, we left the results how they were, as we did not expect them to change with use of SVC.

#### Lipidomics data sets

The differences between GAN and SMOTE/RO were small, but significant, in the case of lipidomics data, with SMOTE/RO performing better. ExpandGAN, following the findings from the microarray experiments, performed significantly worse than GAN, therefore also performing worse than SMOTE/RO. We emphasise the use of less complex data here, compared to the simulation and public microarray datasets, which may have made the data simpler to distinguish between the two classes of the underlying data. As such, the results obtained across this study show evidence towards the beneficial use of the proposed GAN-based methods on more complex datasets, with low numbers of samples, over SMOTE/RO. Importantly, the GAN method resulted in good performance when using the lipidomics datasets, of small difference (although significant) to that of SMOTE/RO, therefore showing evidence that the proposed method does not underperform when data is less complex.

One question that may arise lies with the choice to use the “external” dataset to pre-train the network, rather than its use to balance the “real” data. This choice was made out of the need to make the proposed methodology more flexible. High flexibility has been observed in transfer learning, such as the use of a network pre-trained on image data, transferred to connectome matrices [23]. It was hypothesized that less similar data could be used in place of the “external” data, for datasets that were less widely available. An example would be to use a different cancer for the “external” data, but of the same modality and features as the “real” data. Additionally, three classes were used for the pre-training with the “external” data, when using the lipidomics data, due to the data sharing the same features as the classes the classifier was trained against.

The genes selected for use in the microarray experiments showed evidence for the reflectiveness of the overlying disease. GO and GP results show the significance of TOP2A, which is involved in growth of hepatocellular carcinomas [48]. Additionally, zinc homeostasis is an important prognostic/predictive factor in hepatocellular carcinomas [49], where 6 of the genes (supplementary Table 2) were found to be associated with this pathway.

### Limitations of the study

This study has multiple limitations. Altering the alpha hyperparameters (effect of log L2-matrix norm on generator loss), affected experiments differently, showing positive effects of the performance of the produced GANs on validation if tuned correctly. When using small amounts of data, this aided in the prevention of generated data becoming too similar, ensuring their better distribution, and generalising better over the real distributions of the overlying data. If the value of alpha was set too high, the distributions would become unrealistically wide, causing overlap between the two classes, hindering further model generation from distinguishing between the two classes. The hyperparameter alpha (effect of the L2-matrix norm) did not obviously affect the loss curves during training, therefore, this could only be tuned from the auroc results obtained after the training of the classifiers (validation). In a real scenario, this could not be done, due to bias introduced if tuning using the validation data. Another training dataset could be set aside to tune this hyperparameter, but with the purpose of this method being used when data is limited, this would limit the data further, likely causing reduced performance of the final classifier. Further work would be required to investigate the use of clustering and distance metrics in the training of such networks. We did not evaluate the effect of different sizes of external training data, but the idea is that the more external samples, and the more similar the external data is to the “real” data, the less re-training that needs to be done to make the model specific to the “real” data. This is especially important when there are limited samples in the “real” data. This process can be performed in a systematic way of evaluating the external data.

This study used microarray data, and simulated microarray data. This method requires all samples to have the same variables for training. Additionally, no processing was done to correct for the variations across multiple experiments (ex: batch effect) generated from different studies. Further work should investigate these aspects, to make a more flexible and compatible tool.

We employed feature selection methods in this study for the following purposes: (a) In biomarker studies, the application of feature selection can enhance interpretability and potentially pinpoint more relevant target genes or metabolites, thereby improving overall classification performance. (b) In high-throughput studies such as microarray and RNA sequencing, where the number of expressed genes is substantial (e.g., 20–50 K), not all genes may be associated with the outcome variable of interest. Therefore, selecting “important” features can reduce dimensionality and alleviate computational burden. (c) To individually target specific gene sets, it is essential to identify genes with greater significance (i.e., lower p values).

In our analysis, we utilized filter-based methods relying on univariate selection. In addition, we have used LASSO and EN based methods, which automatically select features based on regularization. Feature selection resulted in large differences between microarray experiment final classification performances, and some differences between lipidomics experiments (although all lipidomics experiments scoring high). Despite these differences, this study was to compare GAN and SMOTE/RO, in which the impact of LASSO and EN feature selection reported little evidence of benefiting some class-balancing techniques over others. Despite this, these experiments demonstrate the importance of selecting an appropriate feature selection method when using the above methods.

## Conclusions

A GAN-based generative method has been designed to improve performance when using very small sample sizes. Evidence of improved performance, in the use of smaller, more complex data, over more naive approaches such as SMOTE and RO were found when training a ‘HistGradientBoostingClassifier’ and SVC on the balanced data, as well as some evidence to suggest improvements in performance compared to a classic WGAN-WP [21].

### Electronic supplementary material

Below is the link to the electronic supplementary material.

Supplementary Material 1

## Data Availability

All the data sets, scripts can be found here: https://github.com/sjcusworth/GAN_Scripts.
