# Supplementary material for: A novel generative adversarial networks modelling for the class imbalance problem in high dimensional omics data
Source: BMC Med Inform Decis Mak. 2024 Mar 28;24:90. doi: 10.1186/s12911-024-02487-2 (PMC10979623; doi:10.1186/s12911-024-02487-2)
Supplement: Supplementary file 1 — Supplementary Material 1 [file 12911_2024_2487_MOESM1_ESM.docx]

**Supplementary tables and figures**

| Supplementary Table 1. **Summary of GO Results**. Summary of GO results obtained from the 135 genes used in the microarray experiments. Top 10 results, by adj.P, are shown.  Gene ontology (GO); adjusted P-value (adj.P). | | | |
| --- | --- | --- | --- |
| Term | Adj.P | Odds.Ratio | Genes |
| RNA binding | 0.013 | 2.744 | TOP2A, SF3B4, PARP1, DARS2, AATF, LSM4, GTPBP4, SMG5, CKAP4, ASS1, LSM2, CS, FBL, STIP1, PSMD4, PRPF3, ZNF207, NUSAP1, CTNNA1, POLR2G, MAPRE1, KPNB1, DAP3 |
| estrogen 2-hydroxylase activity | 0.023 | 99.559 | CYP1A2, CYP3A4 |
| DNA replication origin binding | 0.023 | 22.551 | MCM3, MCM5, MCM6 |
| single-stranded DNA binding | 0.023 | 8.266 | POLR3C, MCM3, MCM5, POLR2G, MCM6 |
| protein kinase binding | 0.023 | 3.471 | TOP2A, GHR, IRAK1, PARP1, DCTN2, PRC1, THY1, MAPRE1, RND3, CDC25B, AURKA |
| 1-alkyl-2-acetylglycerophosphocholine esterase activity | 0.023 | 59.729 | LCAT, PAFAH1B3 |
| galactosidase activity | 0.023 | 59.729 | GBA3, GLA |
| GPI anchor binding | 0.023 | 59.729 | GPAA1, THY1 |
| kinase binding | 0.024 | 3.444 | GHR, IRAK1, PARP1, DCTN2, PRC1, THY1, MAPRE1, RND3, CDC25B, AURKA |
| estrogen 16-alpha-hydroxylase activity | 0.024 | 49.772 | CYP1A2, CYP3A4 |

| Supplementary Table 2. **Summary of WP Results**. Summary of WP results obtained from the 135 genes used in the microarray experiments. Top 10 results, by adj.P, are shown.  WikiPathways Ontology (WP); adjusted P-value (adj.P). | | | |
| --- | --- | --- | --- |
| Term | Adj.P | Odds.Ratio | Genes |
| Zinc homeostasis | 0.000 | 30.752 | MT1M, MT1F, MT1G, MT1X, SLC39A1, MT1E |
| Cell cycle | 0.000 | 11.110 | CDC20, CCNB2, PTTG1, CDK4, MCM3, MCM5, MCM6, CDC25B |
| Retinoblastoma gene in cancer | 0.000 | 13.525 | TOP2A, CCNB2, DNMT1, CDK4, MCM3, MCM6, CDC25B |
| IL-18 signaling pathway | 0.006 | 5.324 | CETP, CCNB2, PYGB, TOMM40, IRAK1, PARP1, RUSC1, PLOD3, FOS |
| TCA Cycle and Deficiency of Pyruvate Dehydrogenase complex (PDHc) | 0.008 | 34.706 | CS, ACLY, PCK1 |
| Amino Acid metabolism | 0.014 | 8.846 | CS, ACLY, DBH, PCK1, ASS1 |
| Copper homeostasis | 0.014 | 12.606 | MT1F, MT1G, MT1X, MT1E |
| Arylamine metabolism | 0.020 | 74.665 | NAT2, CYP1A2 |
| G1 to S cell cycle control | 0.021 | 10.079 | CDK4, MCM3, MCM5, MCM6 |
| Aflatoxin B1 metabolism | 0.021 | 59.729 | CYP1A2, CYP3A4 |


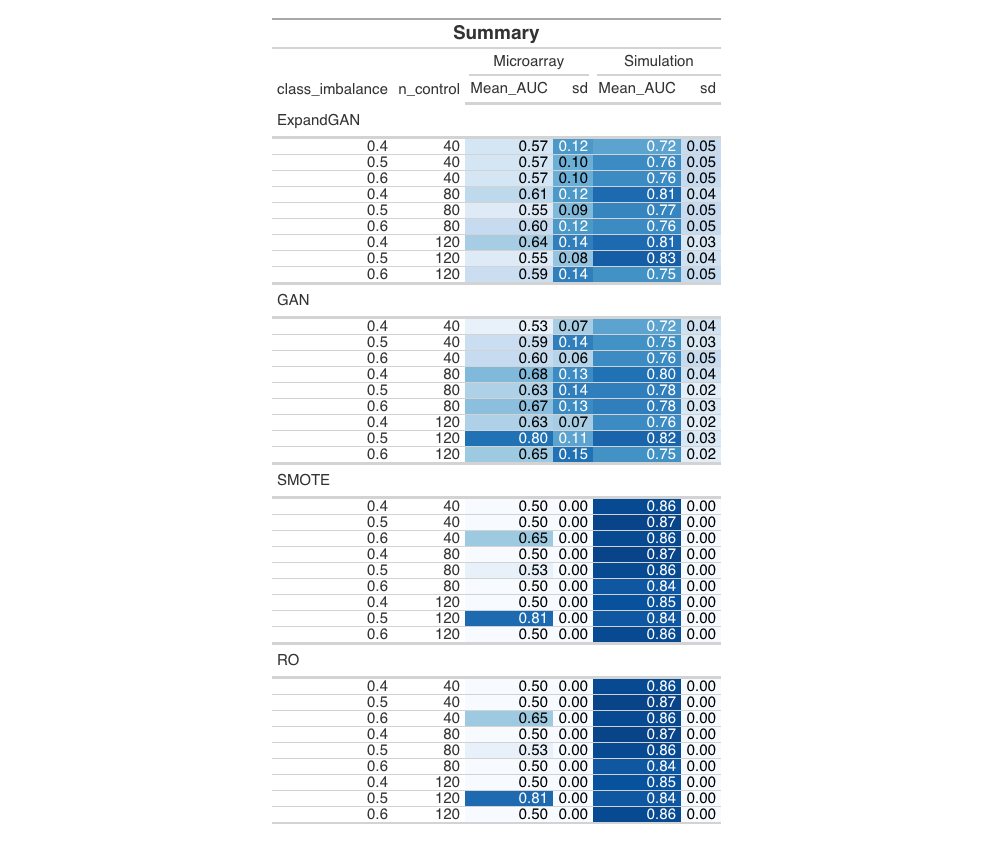


Supplementary Figure 1. **Summary of Validation Scores Across Experiments**. Validation scores are summarised across experiments for both microarray and simulation experiments, grouped by method. Results shown are those using the alpha hyperparameter combination resulting in the greatest mean validation score, and of these, the greatest performing classification method (SVC or ‘HistGradientBoostingClassifier’).

Area under the receiver operating characteristic curve (AUC); standard deviation (sd).


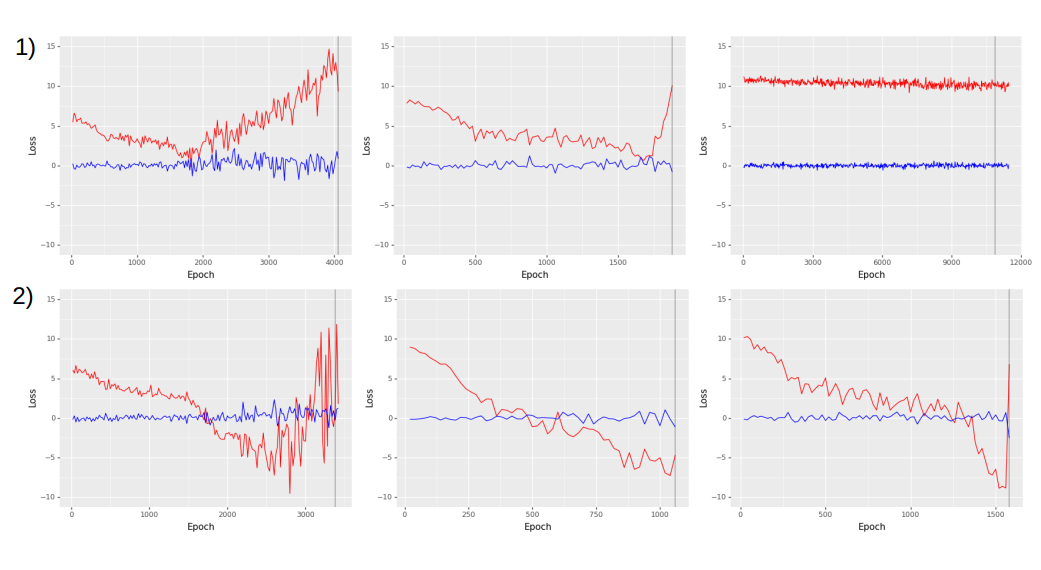


Supplementary Figure 2. **Pre-Training Loss Training Curves**. Loss curves for training with simulation (1) and microarray (2) datasets. Graphs represent training with increasing numbers of layers, 1, 2 and 3 respectively. Vertical, grey line denotes the epoch of which parameters were used in the output model.


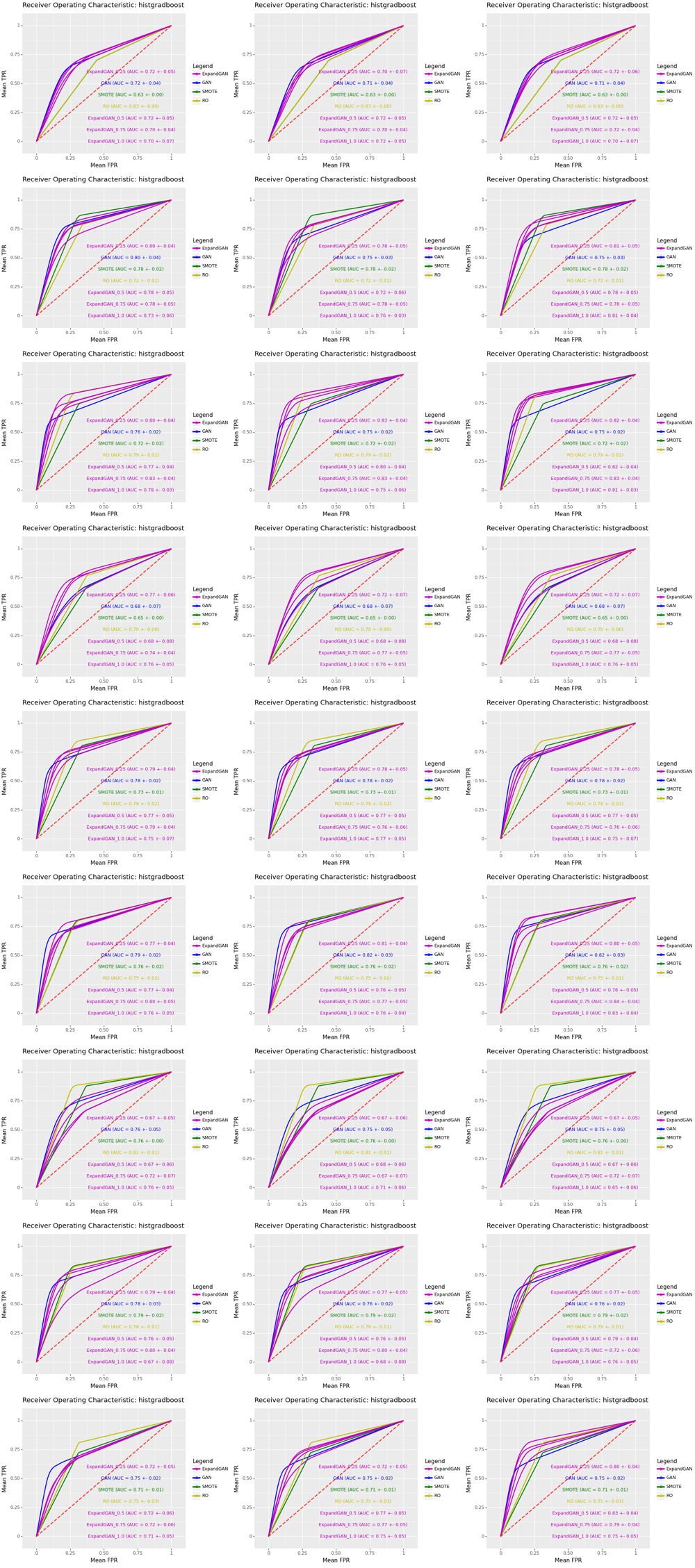


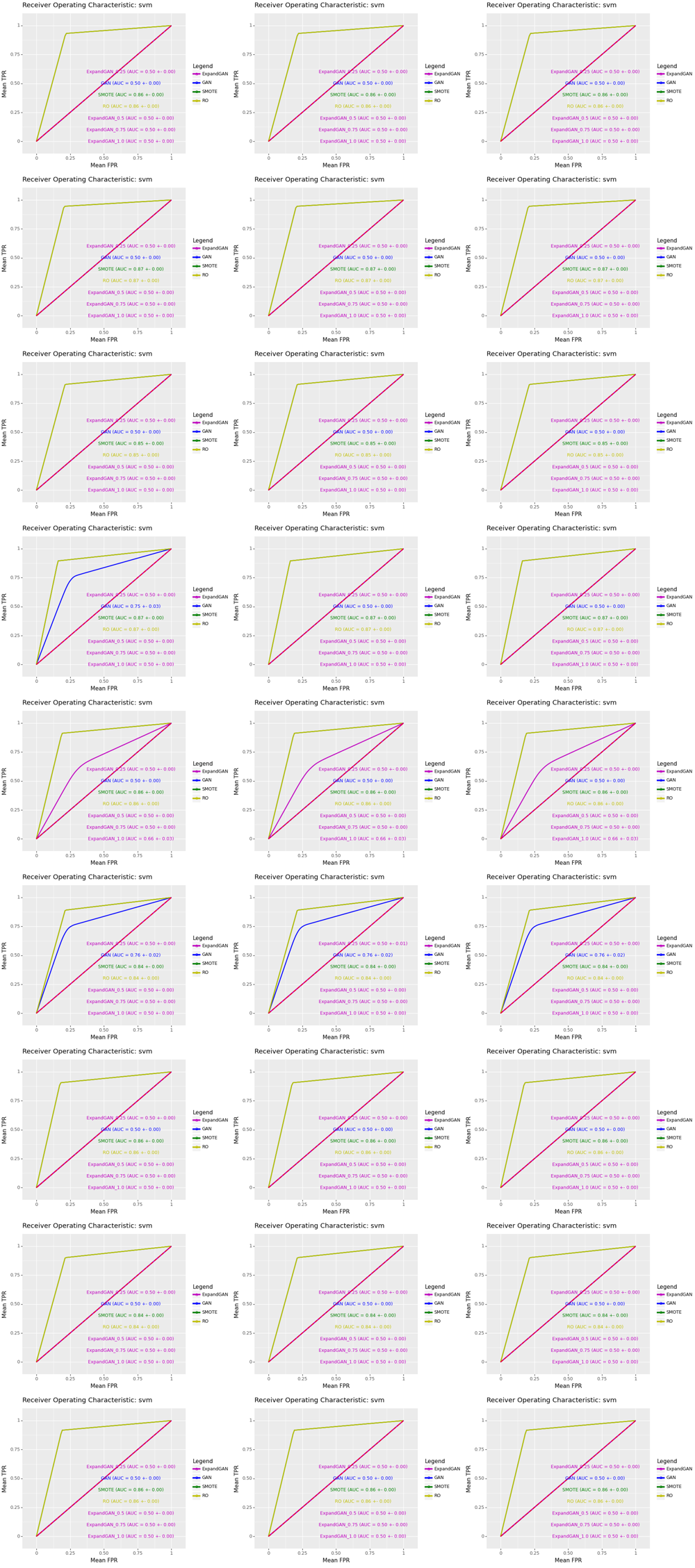


Supplementary Figure 3. **Simulated Microarray ROC Curves**. ROC curves of validated ‘HistGradientBoostingClassifier’ (1) and ‘SVC’ (2) classifiers, when trained on the balanced simulated microarray data. The first column denotes the experiment number of the row. The other columns denote, in order from left to right, alpha hyperparameter combinations of 0/0, 1/0 and 1/1 for underrepresented/overrepresented GANs respectively.

Receiver operating characteristic (ROC), generative adversarial network (GAN) .


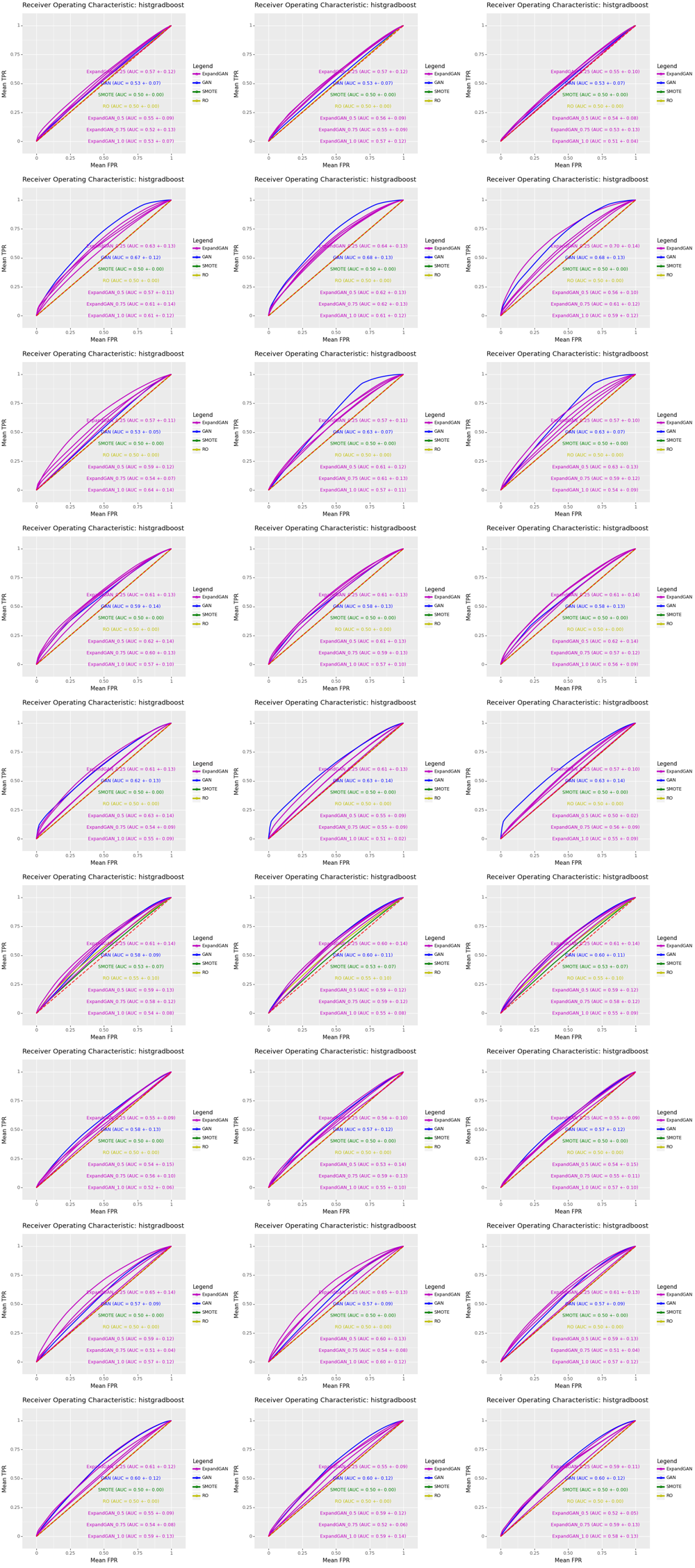


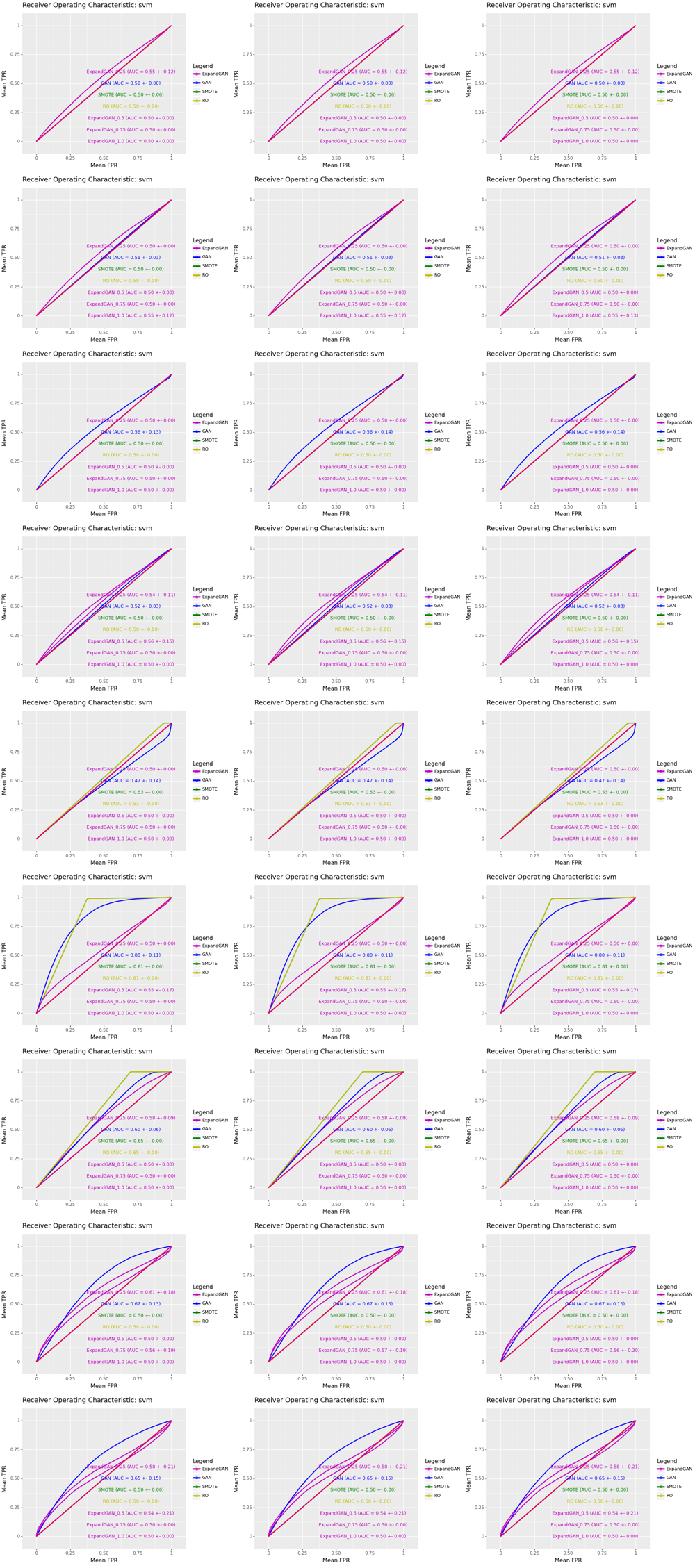


Supplementary Figure 4. **Public Microarray ROC Curves**. ROC curves of validated ‘HistGradientBoostingClassifier’ (1) and ‘SVC’ (2) classifiers, when trained on the balanced public microarray data. The first column denotes the experiment number of the row. The other columns denote, in order from left to right, alpha hyperparameter combinations of 0/0, 1/0 and 1/1 for underrepresented/overrepresented GANs respectively.

Receiver operating characteristic (ROC), generative adversarial network (GAN).


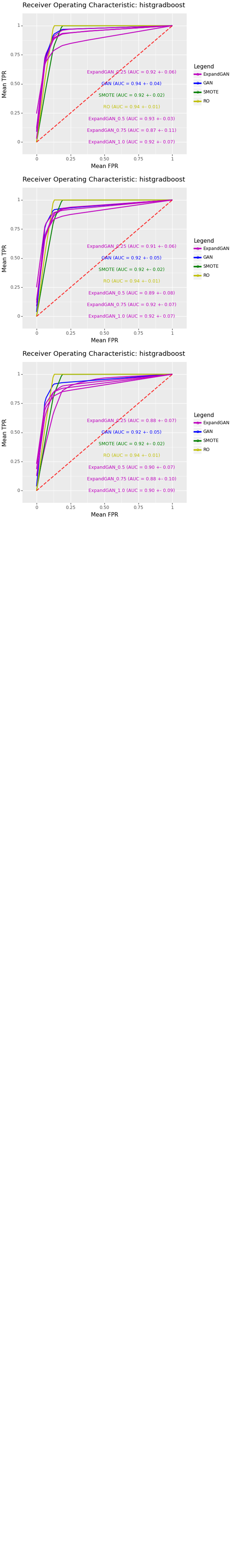


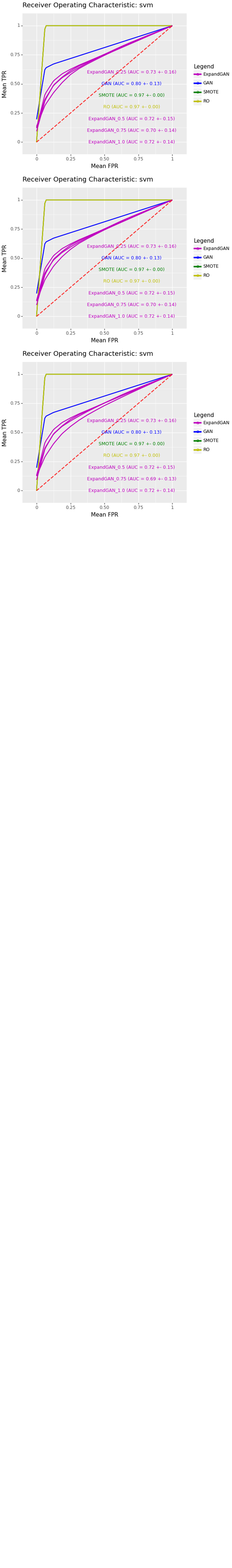


Supplementary Figure 5. **Public Lipidomics ROC Curves**. ROC curves of validated ‘HistGradientBoostingClassifier’ (1) and ‘SVC’ (2) classifiers, when trained on the balanced public microarray data. The first column denotes the experiment number of the row. The other columns denote, in order from left to right, alpha hyperparameter combinations of 0/0, 1/0 and 1/1 for underrepresented/overrepresented GANs respectively.

Receiver operating characteristic (ROC), generative adversarial network (GAN).


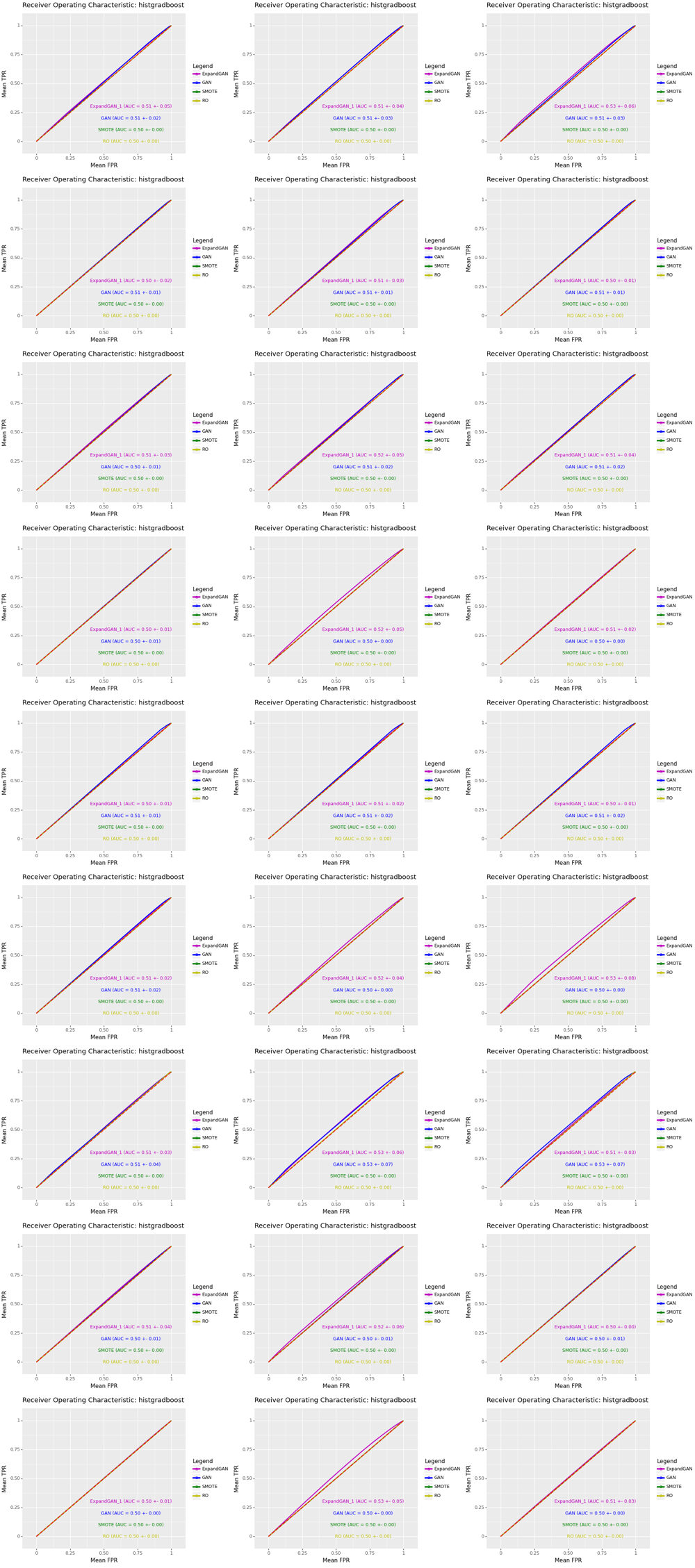


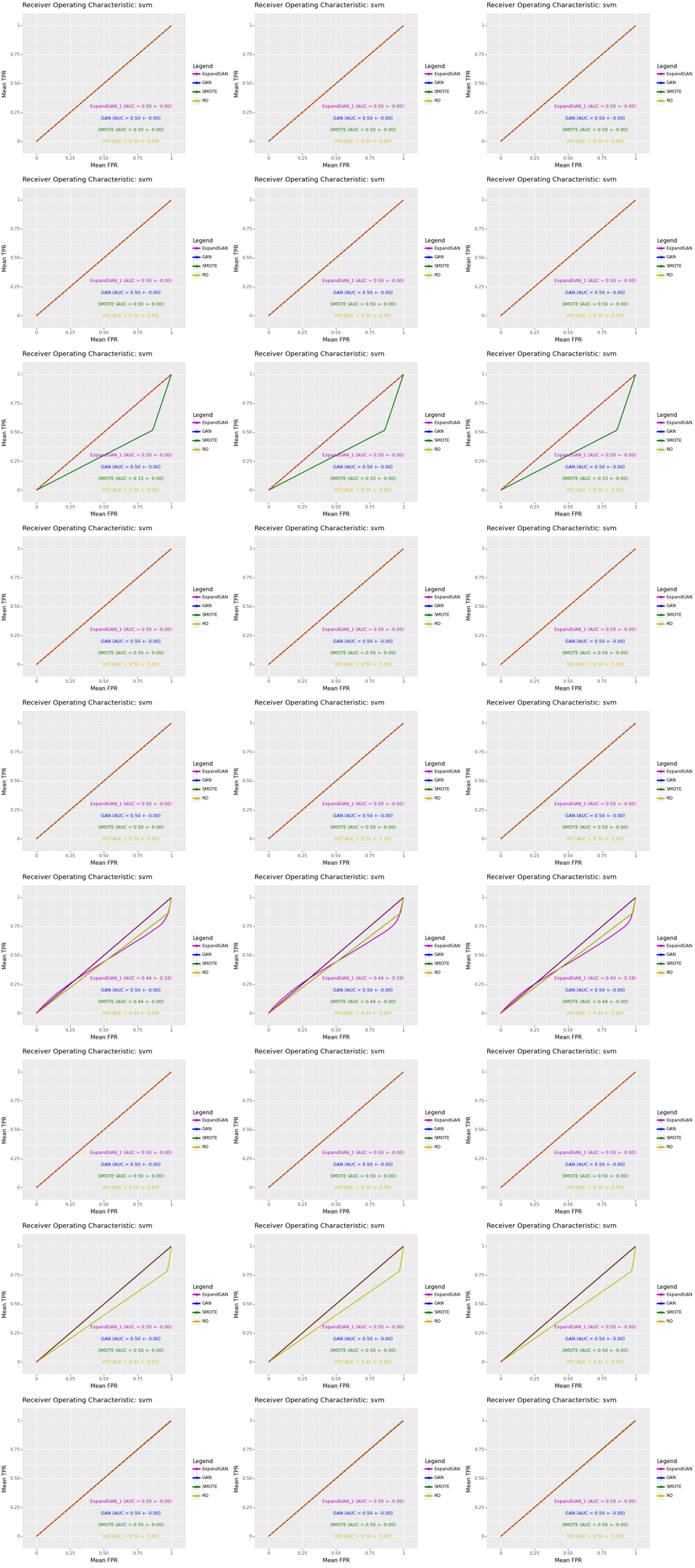


Supplementary Figure 6. **Public Microarray (Lasso Feature Selection) ROC Curves**. ROC curves of validated ‘HistGradientBoostingClassifier’ (1) and ‘SVC’ (2) classifiers, when trained on the balanced public microarray data. The first column denotes the experiment number of the row. The other columns denote, in order from left to right, alpha hyperparameter combinations of 0/0, 1/0 and 1/1 for underrepresented/overrepresented GANs respectively.

Receiver operating characteristic (ROC), generative adversarial network (GAN).


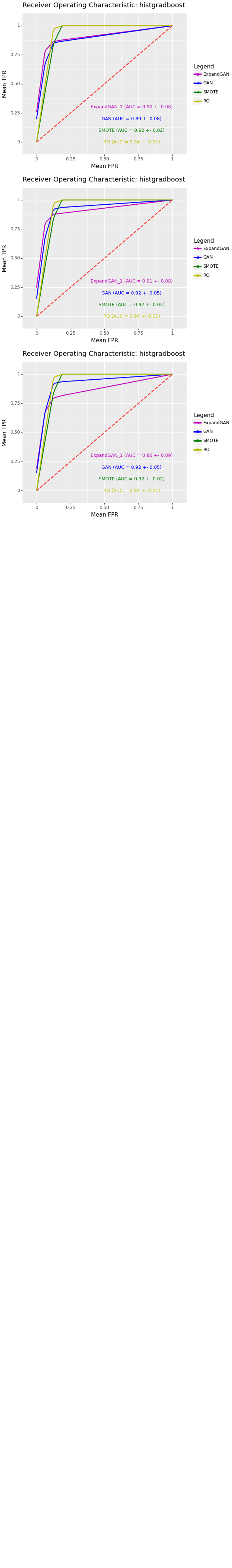


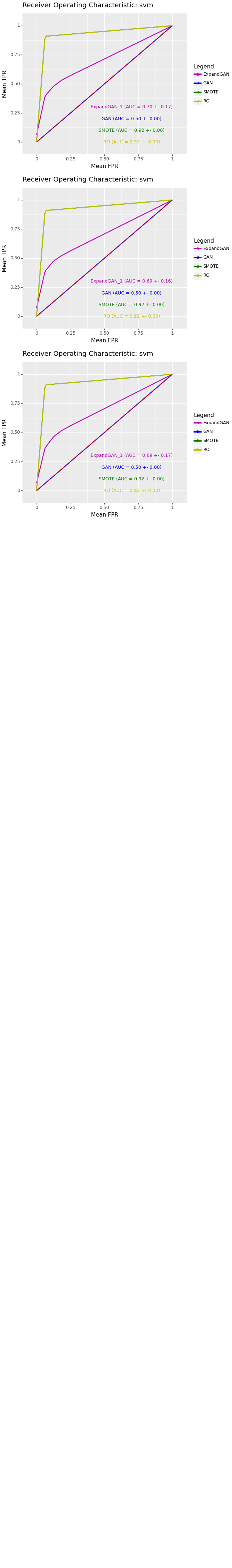


Supplementary Figure 7. **Public Lipidomics (Lasso Feature Selection) ROC Curves**. ROC curves of validated ‘HistGradientBoostingClassifier’ (1) and ‘SVC’ (2) classifiers, when trained on the balanced public microarray data. The first column denotes the experiment number of the row. The other columns denote, in order from left to right, alpha hyperparameter combinations of 0/0, 1/0 and 1/1 for underrepresented/overrepresented GANs respectively.

Receiver operating characteristic (ROC), generative adversarial network (GAN).
